# Proteomic profiling of the rat hypothalamus

**DOI:** 10.1186/1477-5956-10-26

**Published:** 2012-04-20

**Authors:** Amanda P Pedroso, Regina L H Watanabe, Kelse T Albuquerque, Mônica M Telles, Maria C C Andrade, Juliana D Perez, Maísa M Sakata, Mariana L Lima, Debora Estadella, Cláudia M O Nascimento, Lila M Oyama, José C Rosa, Dulce E Casarini, Eliane B Ribeiro

**Affiliations:** 1 Department of Physiology, Division of Nutrition Physiology, Universidade Federal de São Paulo, UNIFESP, São Paulo, SP, Brazil; 2 Department of Biological Sciences, Universidade Federal de São Paulo, UNIFESP, São Paulo, SP, Brazil; 3 Department of Medicine, Division of Nephrology, Universidade Federal de São Paulo, UNIFESP, São Paulo, SP, Brazil; 4 Department of Microbiology, Immunology and Parasitology, Universidade Federal de São Paulo, UNIFESP, São Paulo, SP, Brazil; 5 Centro de Química de Proteínas, FMRP, Universidade de São Paulo, Ribeirão Preto, SP, Brazil; 6 Departamento de Fisiologia, Universidade Federal de São Paulo – Escola Paulista de Medicina, Rua Botucatu 862 2° andar, Vila Clementino, 04023-062, São Paulo, SP, Brazil

**Keywords:** Hypothalamus, Proteome, 2-D electrophoresis, Mass spectrometry, Nutrition, Obesity, Rat

## Abstract

**Background:**

The hypothalamus plays a pivotal role in numerous mechanisms highly relevant to the maintenance of body homeostasis, such as the control of food intake and energy expenditure. Impairment of these mechanisms has been associated with the metabolic disturbances involved in the pathogenesis of obesity. Since rodent species constitute important models for metabolism studies and the rat hypothalamus is poorly characterized by proteomic strategies, we performed experiments aimed at constructing a two-dimensional gel electrophoresis (2-DE) profile of rat hypothalamus proteins.

**Results:**

As a first step, we established the best conditions for tissue collection and protein extraction, quantification and separation. The extraction buffer composition selected for proteome characterization of rat hypothalamus was urea 7 M, thiourea 2 M, CHAPS 4%, Triton X-100 0.5%, followed by a precipitation step with chloroform/methanol. Two-dimensional (2-D) gels of hypothalamic extracts from four-month-old rats were analyzed; the protein spots were digested and identified by using tandem mass spectrometry and database query using the protein search engine MASCOT. Eighty-six hypothalamic proteins were identified, the majority of which were classified as participating in metabolic processes, consistent with the finding of a large number of proteins with catalytic activity. Genes encoding proteins identified in this study have been related to obesity development.

**Conclusion:**

The present results indicate that the 2-DE technique will be useful for nutritional studies focusing on hypothalamic proteins. The data presented herein will serve as a reference database for studies testing the effects of dietary manipulations on hypothalamic proteome. We trust that these experiments will lead to important knowledge on protein targets of nutritional variables potentially able to affect the complex central nervous system control of energy homeostasis.

## Background

The hypothalamus plays a pivotal role in numerous mechanisms highly relevant to maintenance of body homeostasis. By exerting important control of autonomic and endocrine functions, it affects aspects as critical as body temperature, food intake, energy expenditure, water intake, and intermediary metabolism.

Concerning the control of body energy homeostasis, the hypothalamus exerts an integrative influence, as it receives neural, hormonal, and metabolic signals that inform about body energy status. These inputs modify the activity of hypothalamic anabolic and catabolic neurons, whose neuropeptide products stimulate or inhibit food intake, respectively. Disturbances of these control mechanisms have been associated with the pathogenesis of obesity [1-4].

In this study, we used two-dimensional electrophoresis (2-DE) to separate proteins expressed in the rat hypothalamus. The separated proteins were analysed by matrix-assisted laser desorption ionization time-of-flight mass spectrometry (MALDI-TOF/TOF MS) and the obtained spectra used to determine protein identity.

Proteomic analysis is relatively new in nutrition studies and its potential to contribute to this field has been emphasized [5,6]. Few studies have used proteomic technology in hypothalamus. It has recently been reported that the hypothalamic expression of Ubiquitin and Ubiquitin carboxyl-terminal esterase L1 (UCHL-1), both components of Ubiquitin-proteasome system, was altered in diet-induced obese rats when compared with diet-resistant rats [7]. Chronic circadian desynchronization in rats led to increased food intake and body weight while affecting expression of five hypothalamic proteins, some of which are involved in carbohydrate metabolism and the Krebs cycle [8]. In another study, estradiol replacement in ovariectomized rats was shown to regulate expression of 29 proteins in ventromedial hypothalamus, some of which are related to neuronal plasticity [9].

The above considerations suggest that proteomic studies of hypothalamic tissue, in the context of nutritional aspects, may provide relevant information not only on physiological control of food intake and energy homeostasis but also on the pathophysiology of obesity and other feeding disturbances. Since the rat hypothalamus is poorly characterized by proteomic strategies, this study focused on identifying proteins expressed in hypothalamus of normal male *Wistar* rats.

## Results and discussion

Reported advantages of protein separation by 2-DE for proteomic analysis include robustness, reproducibility, statistical confidence and ability to separate complete proteins, while sample preparation has been considered a main factor still contributing to variability [10].

In this study, a two-dimensional profile of rat hypothalamus proteins was constructed. Considering that the experimental protocol is a highly relevant aspect in proteomic studies, as it ensures satisfactory resolution and proteome representation [11,12], experiments were performed aimed at establishing the best conditions for tissue withdrawal and protein extraction, quantification and separation. These results are not shown and we comment on these findings below.

Initially, 5 different extraction buffers were used (Table 1) and the resulting 2-D maps were compared for gel resolution and spot counting. Although all buffers were efficient for hypothalamic protein extraction, the best protein recovery was achieved with buffer 3, regardless the presence of ASB-14 or DTT (data not shown). The presence of both urea and thiourea was probably effective, because this combination has been shown to increase membrane and nuclear proteins solubilisation, even of proteins prone to undergo precipitation during isoelectric focusing [13].

**Table 1:** Sample preparation procedures and buffers composition

		**1**	**2**	**3**	**3 + DTT**	**3 + ASB-14**
**Extraction**	Tris–HCl pH 7,6	40 mM	-	-	-	-
	Tris	-	20 mM	-	-	-
	Urea	-	9 M	7 M	7 M	7 M
	Tiourea	-	-	2 M	2 M	2 M
	EDTA	-	1 mM	-	-	-
	CHAPS (w/v)	2%	4%	4%	4%	4%
	Triton X-100 (v/v)	-	-	0.5%	0.5%	0.5%
	DTT	-	10 mM	-	100 mM	-
	ASB-14 (w/v)	-	-	-	-	2%
**Precipitation**		No	No	Yes	Yes	Yes
**Rehydration**	Tiourea	2 M	-		2 M	
	Urea	7 M	9 M		7 M	
	DTT	50 mM	18 mM		100 mM	
	CHAPS (w/v)	2%	2%		4%	
	Triton x-100 (v/v)	-	-		0.5%	
	IPG Buffer pH 3-10 (v/v)	0.5%	2%		0.2%	
	Bromofenol blue	traces	traces		traces	

One aspect tested in the present study was the importance of the presence of DTT as reducing agent in the extraction buffer. DTT, which breaks disulfide bonds, has been used in numerous proteomic studies to favour protein solubility in the extraction solution [14]. Indeed, proteome analysis of rat and mice brain regions has used DTT, in concentrations ranging from 10 to 100 mM [8,9,15-17]. Because we failed to find differences in protein recovery by the addition of 100 mM DTT to buffer 3, it is suggested that the presence of 100 mM DTT in the rehydration buffer was sufficient to efficiently reduce hypothalamic proteins.

The addition of 2% ASB-14 to CHAPS-containing extraction buffer has been shown to improve solubilization of human frontal cortex proteins, when no sample precipitation was performed [18]. In the present experiments with rat hypothalamus, we failed to obtain higher protein recovery by performing precipitation of samples extracted in the presence of ASB-14. We suggest that the precipitation procedure may have interfered with protein recovery extracted in buffer containing ASB-14. Other authors reporting beneficial effects of ASB-14 have also omitted the precipitation step [19,20].

We chose to include the precipitation step in the present study as this procedure is believed to improve 2-DE quality by eliminating contaminating species (lipids, nucleic acids, and salts), although it may lead to incomplete protein recovery [21,22]. Among different precipitation methods, chloroform/methanol, as used in the present study, has been recently shown to result in better protein yields from rat hippocampus, amygdala, frontal cortex and striatum [23].

Given all the above considerations, extraction buffer 3 was selected for the proteome characterization of rat hypothalamus. Six extracts from six different four-month-old rats were focused with pH 3–10 strips, in two independent experiments, and the 2-DE gels were analyzed. Each rat sample was analyzed individually, with no tissue pooling. Details of isoelectric focusing and gel separation are given in the Methods section. A high degree of identity among the gels was confirmed by a high scatter plot correlation coefficient (>85%).

**Figure 1:**
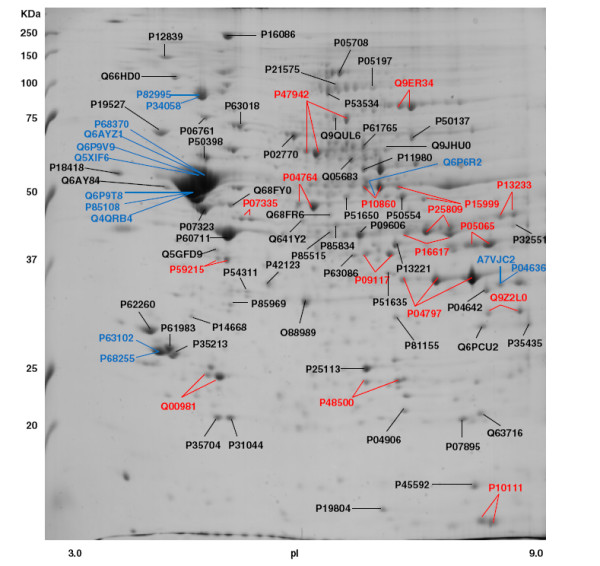
**Representative 2-D gel of rat hypothalamus stained with Coomassie Blue G-250.** The proteins identified are designated with their Swiss-Prot accession numbers. Multiple lines originating from the same Accession Number indicate that the same protein was identified in different spots. Multiple lines originating from the same spot indicate that more than one protein was identified in that spot. Detailed information about the proteins are found in Table 2.

Figure 1 shows a representative 2-D gel with molecular weight range from 10 to 250 KDa and pI from 3–9. The number of spots obtained was 234 ± 6, of which 137 were analyzed by mass spectrometry. All analyzed spots were present in all six gels. Table 2 shows the Swiss-Prot Accession Numbers (available at http://www.expasy.ch/sprot), abbreviated and full protein names, theoretical MW and pI values, and mass spectrometry data. Only statistically significant Mascot score results (p < 0.05) were included.

**Table 2:** Identified proteins from the rat hypothalamus

**Name/** **Acession** **Number**^**a**^	**Full name**	**MW (Da)/** **pI**	**Matches/** **SC**^**b**^	**PS**^**c**^	**Peptides**	**IS**^**d**^
G3P_RAT/P04797	Glyceraldehyde-3-phosphate dehydrogenase	36090/ 8.14	4(4)/ 17%	274	M.VKVGVNGFGR.I R.VPTPNVSVVDLTCR.L K.LISWYDNEYGYSNR.V K.AENGKLVINGKPITIFQER.D	47 97 92 38
			4(2)/ 15%	108	M.VKVGVNGFGR.I R.VPTPNVSVVDLTCR.L K.LVINGKPITIFQER.D K.LISWYDNEYGYSNR.V	46 27 16 19
			3(2)/ 11%	106	M.VKVGVNGFGR.I R.VPTPNVSVVDLTCR.L K.LISWYDNEYGYSNR.V	38 19 49
SSDH_RAT/P51650	Succinate-semialdehyde dehydrogenase, mitochondrial	56723/ 8.35	1(1)/ 1%	44	R.AAYDAFSSWK.E	44
DHE3_RAT/P10860	Glutamate dehydrogenase 1, mitochondrial	61719/ 8.05	4(3)/ 10%	204	K.MVEGFFDR.G + Oxidation (M) R.DDGSWEVIEGYR.A K.HGGTIPVVPTAEFQDR.I K.IIAEGANGPTTPEADKIFLER.N	17 60 57 70
			5(5)/ 14%	216	R.DDGSWEVIEGYR.A K.DIVHSGLAYTMER.S + Oxidation (M) K.HGGTIPVVPTAEFQDR.I K.KGFIGPGIDVPAPDMSTGER.E + Oxidation (M) K.IIAEGANGPTTPEADKIFLER.N	57 41 53 40 27
AK1A1_RAT/P51635	Alcohol dehydrogenase [NADP+]	36711/ 6.84	2(0)/ 8%	29	R.DAGHPLYPFNDPY.- R.GLEVTAYSPLGSSDR.A	20 9
CN37_RAT/P13233	2',3'-cyclic-nucleotide 3'-phosphodiesterase	47638/ 9.03	4(3)/ 7%	169	K.AIFTGYYGK.G R.ADFSEEYKR.L K.ATGAEEYAQQDVVR.R K.ATGAEEYAQQDVVRR.S	51 49 45 23
			4(4)/ 7%	175	K.AIFTGYYGK.G R.ADFSEEYKR.L K.ATGAEEYAQQDVVR.R K.ATGAEEYAQQDVVRR.S	69 40 36 30
DPYL2_RAT/P47942	Dihydropyrimidinase-related protein 2	62638/ 5.95	5(4)/ 14%	160	R.KPFPDFVYKR.I K.IVLEDGTLHVTEGSGR.Y K.DNFTLIPEGTNGTEER.M R.NLHQSGFSLSGAQIDDNIPR.R R.DIGAIAQVHAENGDIIAEEQQR.I	20 31 46 25 39
			3(1)/ 6%	67	K.VFNLYPR.K R.KPFPDFVYKR.I R.ISVGSDADLVIWDPDSVK.T	19 36 12
			4(3)/ 5%	164	K.VFNLYPR.K R.KPFPDFVYKR.I K.THNSALEYNIFEGMECR.G K.THNSALEYNIFEGMECR.G + Oxidation (M)	37 69 58 (15)
DPYL5_RAT/Q9JHU0	Dihydropyrimidinase-related protein 5	62071/ 6.60	4(0)/ 7%	45	K.IPHGVSGVQDR.M R.DSELYQVFHACR.D R.GRVVYENGVFMCAEGTGK.F R.GRVVYENGVFMCAEGTGK.F + Oxidation (M)	4 19 (8) 23
UCHL1_RAT/Q00981	Ubiquitin carboxyl-terminal hydrolase isozyme L1	25165/ 5.14	4(2)/ 22%	157	K.QFLSETEK.L K.QFLSETEKLSPEDR.A -.MQLKPMEINPEMLNK.V + 3 Oxidation (M) R.MPFPVNHGASSEDSLLQDAAK.V + Oxidation (M)	52 82 9 16
			2(1)/ 15%	52	K.QFLSETEKLSPEDR.A R.MPFPVNHGASSEDSLLQDAAK.V + Oxidation (M)	38 16
ATPG_RAT/P35435	ATP synthase subunit gamma, mitochondrial	30229/ 8.87	2(1)/ 8%	73	R.VYGTGSLALYEK.A R.THSDQFLVSFK.D	63 10
VATE1_RAT/Q6PCU2	V-type proton ATPase subunit E 1	26169/ 8.44	4(2)/ 14%	102	R.GALFGANANR.K R.GALFGANANRK.F K.AEEEFNIEKGR.L K.IQMSNLMNQAR.L + 2 Oxidation (M)	15 31 45 13
GABT_RAT/P50554	4-aminobutyrate aminotransfe- rase, mitochondrial	57161/ 8.15	4(2)/ 7%	124	K.TIFMWYR.S K.VDFEFDYDGPLMK.T + Oxidation (M) R.GTFCSFDTPDEAIR.N R.GRGTFCSFDTPDEAIR.N	6 49 52 17
AATC_RAT/P13221	Aspartate aminotransferase cytoplasmic	46628/ 6.73	4(4)/ 13%	149	R.ITWSNPPAQGAR.I R.TDDSQPWVLPVVR.K R.SCASQLVLGDNSPALR.E R.IVATTLSNPELFKEWK.G	36 40 38 35
NDKB_RAT/P19804	Nucleoside diphosphate kinase B	17386/ 6.92	2(0)/ 14%	32	K.DRPFFPGLVK.Y R.TFIAIKPDGVQR.G	12 19
KPYM_RAT/P11980	Pyruvate kinase isozymes M1/M2	58294/ 6.63	4(2)/ 9%	85	R.LLFEELAR.A R.YRPRAPIIAVTR.N K.CLAAALIVLTESGR.S R.RFDEILEASDGIMVAR.G + Oxidation (M)	43 4 30 10
HXK1_RAT/P05708	Hexokinase-1	103540/ 6.29	6(2)/ 5%	125	K.EGLLFEGR.I K.MHPQYSR.R + Oxidation (M) R.ITPELLTR.G K.FLSQIESDR.L R.QIEETLAHFR.L K.MISGMYLGEIVR.N + 2 Oxidation (M)	28 17 11 23 53 2
PGK1_RAT/P16617	Phosphoglycerate kinase 1	44909/ 8.02	3(2)/ 11%	70	K.LGDVYVNDAFGTAHR.A K.ALESPERPFLAILGGAK.V K.VLNNMEIGTSLYDEEGAK.I + oxidation (M)	38 6 27
			2(2)/ 7%	76	K.LGDVYVNDAFGTAHR.A K.VLNNMEIGTSLYDEEGAK.I + Oxidation (M)	26 50
KCRB_RAT/P07335	Creatine kinase B-type	42983/ 5.39	5(4)/ 18%	318	R.GFCLPPHCSR.G K.VLTPELYAELR.A K.LAVEALSSLDGDLSGR.Y K.TFLVWINEEDHLR.V R.GTGGVDTAAVGGVFDVSNADR.L	44 56 67 22 131
			2(1)/ 5%	52	R.MGGYQPSDEHK.T + oxidation (HW) K.VLTPELYAELR.A	7 45
KCRU_RAT/P25809	Creatine kinase U-type	47398/ 8.72	5(2)/ 12%	102	R.GWEFMWNER.L + oxidation (M) R.LYPPSAEYPDLR.K R.LYPPSAEYPDLRK.H R.VVVDALSGLKGDLAGR.Y K.SFLIWVNEEDHTR.V	19 28 17 11 32
			5(3)/ 12%	164	R.GWEFMWNER.L + Oxidation (M) R.LYPPSAEYPDLR.K R.LYPPSAEYPDLRK.H K.SFLIWVNEEDHTR.V R.LGYILTCPSNLGTGLR.A	18 50 17 56 25
ENOG_RAT/P07323	Gamma-enolase	47510/ 5.03	5(2)/ 14%	98	R.FAGHNFRNPSVL.- K.MVIGMDVAASEFYR.D + Oxidation (M) K.MVIGMDVAASEFYR.D + 2 Oxidation (M) R.AAVPSGASTGIYEALELR.D K.LAMQEFMILPVGAESFR.D + 2 Oxidation (M)	18 (6) 17 41 26
ALDOC_RAT/P09117	Fructose-biphosphate aldolase C	39658/ 6.67	5(4)/ 20%	264	R.ALQASALSAWR.G R.DNAGAATEEFIKR.A M.PHSYPALSAEQKK.E R.CSLPRPWALTFSYGR.A K.YEGSGDGGAAAQSLYVANHAY.-	59 84 75 8 38
			4(1)/ 15%	104	K.ELSDIALR.I R.DNAGAATEEFIKR.A R.LSQIGVENTEENRR.L K.YEGSGDGGAAAQSLYVANHAY.-	19 55 24 9
ENOA_RAT/P04764	Alpha-enolase	47440/ 6.16	6(4)/ 17%	233	R.SFRNPLAK.- K.LAQSNGWGVMVSHR.S K.LAQSNGWGVMVSHR.S + Oxidation (M) R.AAVPSGASTGIYEALELR.D K.LAMQEFMILPVGASSFR.E + 2 Oxidation (M) K.AGYTDQVVIGMDVAASEFYR.A + Oxidation (M)	36 (22) 24 99 31 46
			2(1)/ 8%	60	R.AAVPSGASTGIYEALELR.D K.AGYTDQVVIGMDVAASEFYR.A + Oxidation (M)	19 41
ALDOA_RAT/P05065	Fructose-biphosphate aldolase A	39783/ 8.31	4(2)/ 14%	113	M.PHPYPALTPEQK.K M.PHPYPALTPEQKK.E K.FSNEEIAMATVTALRR.T + Oxidation (M) K.YTPSGQSGAAASESLFISNHAY.	40 51 22 6
			2(1)/9%	54	M.PHPYPALTPEQKK.E K.YTPSGQSGAAASESLFISNHAY.	40 17
DCE2_RAT/Q05683	Glutamate decarboxylase 2	66215 /6.45	1(1)/ 3%	27	K.LCALLYGDSEKPAESGGSVTSR.A	27
PYGB_RAT/P53534	Glycogen phosphorylase, brain form (fragment)	96854/ 6.24	5(1)/ 6%	98	R.VIFLENYR.V R.DYFFALAHTVR.D R.HLEIIYAYNQR.H R.VLYPNDNFFEGK.E K.ARPEYMLPVHFYGR.V + oxidation (HW)	5 25 44 22 8
TPIS_RAT/P48500	Triosephosphate isomerase	27345/ 6.89	4(3)/ 14%	115	K.FFVGGNWK.M R.KFFVGGNWK.M R.IIYGGSVTGATCK.E K.DLGATWVVLGHSER.R	45 8 29 32
			5(2)/ 20%	147	K.FFVGGNWK.M R.KFFVGGNWK.M R.IIYGGSVTGATCK.E K.DLGATWVVLGHSER.R R.RHIFGESDELIGQK.V	49 14 59 10 15
GLNA_RAT/P09606	Glutamine synthetase	42982/ 6.64	5(2)/ 16%	133	K.RHQYHIR.A K.IQLMYIWVDGTGEGLR.C + Oxidation (M) R.RPSANCDPYAVTEAIVR.T R.LTGFHETSNINDFSAGVANR.S R.RLTGFHETSNINDFSAGVANR.S	24 43 19 40 10
CALR_RAT/P18418	Calreticulin	48137/ 4.33	3(2)/ 9%	197	K.EQFLDGDAWTNR.W K.HEQNIDCGGGYVK.L K.IKDPDAAKPEDWDER.A	102 13 82
SCRN1_RAT/Q6AY84	Secernin-1	46994/ 4.73	2(2)/ 7%	88	K.VECTYISIDQVPR.T R.SSPCIHYFTGTPDPSR.S	49 39
IMPCT_RAT/Q5GFD9	Protein IMPACT	36314/ 5.07	2(1)/ 10%	37	K.SLEEIYMK.N + Oxidation (M) K.ISESAPEAEELPPIAHGAPITDRR.S	2 35
PGAM1_RAT/P25113	Phosphoglycerate mutase 1	28928/ 6.67	3(2)/ 18%	121	R.HGESAWNLENR.F R.ALPFWNEEIVPQIK.E R.SYDVPPPPMEPDHPFYSNISK.D + Oxidation (M)	59 44 21
VDAC1_RAT/Q9Z2L0	Voltage-dependent anion-selective channel protein 1	30851/ 8.62	3(1)/ 8%	66	R.WTEYGLTFTEK.W K.YQVDPDACFSAK.V K.YRWTEYGLTFTEK.W	40 16 12
			3(1)/ 12%	86	R.VTQSNFAVGYK.T R.WTEYGLTFTEK.W K.YQVDPDACFSAK.V	13 24 51
VDAC2_RAT/P81155	Voltage-dependent anion- selective channel protein 2	32353/ 7.44	3(2)/ 17%	121	R.SNFAVGYR.T K.VNNSSLIGVGYTQTLRPGVK.L R.TGDFQLHTNVNNGTEFGGSIYQK.V	41 71 9
ALBU_RAT/P02770	Serum albumin	70682/ 6.09	3(2)/ 7%	83	K.LGEYGFQNAVLVR.Y K.LRDNYGELADCCAK.Q K.AADKDNCFATEGPNLVAR.S	35 19 29
NSF_RAT/Q9QUL6	Vesicle-fusing ATPase	83170/ 6.55	3(0)/ 4%	40	K.GILLYGPPGCGK.T K.NFSGAELEGLVR.A R.QSIINPDWNFEK.M	25 6 12
EF1G_RAT/Q68FR6	Elongation factor 1-gamma	50371/ 6.31	2(0)/ 4%	38	K.AKDPFAHLPK.S K.STFVLDEFKR.K	14 24
1433G_RAT/P61983	14-3-3 protein gamma	28456/ 4.80	1(1)/ 5%	88	K.NVTELNEPLSNEER.N	88
1433E_RAT/P62260	14-3-3 protein épsilon	29326/ 4.63	3(3)/ 9%	142	R.YLAEFATGNDR.K R.YLAEFATGNDRK.E K.VAGMDVELTVEER.N + Oxidation (M)	36 77 29
1433B_RAT/P35213	14-3-3 protein beta/alpha	28151/ 4.81	3(0)/ 13%	48	R.NLLSVAYK.N K.DSTLIMQLLR.D + Oxidation (M) K.AVTEQGHELSNEER.N	16 15 18
1433Z_RAT/P63102	14-3-3 protein zeta/delta	27925/ 4.73	3(2)/ 16%	148	R.NLLSVAYK.N K.SVTEQGAELSNEER.N K.GIVDQSQQAYQEAFEISK.K	25 92 31
1433T_RAT/P68255	14-3-3 protein theta	28046/ 4.69	2(1)/ 8%	76	R.NLLSVAYK.N K.AVTEQGAELSNEER.N	25 51
PEBP1_RAT/P31044	Phosphatidylethanolamine- binding protein 1	20902/ 5.48	3(2)/ 22%	149	K.FKVESFR.K R.VDYGGVTVDELGK.V K.YHLGAPVAGTCFQAEWDDSVPK.L	39 92 18
EF2_RAT/P05197	Elongation factor 2	96192/ 6.41	3(1)/ 5%	74	M.VNFTVDQIR.A K.AYLPVNESFGFTADLR.S K.ARPFPDGLAEDIDKGEVSAR.Q	42 21 11
GNAO_RAT/P59215	Guanine nucleotide-binding protein G(o) subunit alpha	40613/ 5.34	5(2)/ 15%	114	K.YYLDSLDR.I R.AMDTLGVEYGDKER.K + Oxidation (M) R.IGAADYQPTEQDILR.T R.MEDTEPFSAELLSAMMR.L + 2 Oxidation (M) R.MEDTEPFSAELLSAMMR.L + 3 Oxidation (M)	17 29 59 (5) 8
			3(1)/ 12%	66	R.AMDTLGVEYGDKER.K + Oxidation (M) R.IGAADYQPTEQDILR.T R.MEDTEPFSAELLSAMMR.L + 3 Oxidation (M)	18 45 3
GBB1_RAT/P54311	Guanine nucleotide-binding protein G(I)/G(S)/G(T) subunit beta-1	38151/ 5.60	3(2)/ 7%	76	K.IYAMHWGTDSR.L K.IYAMHWGTDSR.L + Oxidation (M) R.ELAGHTGYLSCCR.F	43 (18) 33
GDIA_RAT/P50398	Rab GDP dissociation inhibitor alpha	51074/ 5.00	5(4)/ 16%	195	R.IKLYSESLAR.Y R.GRDWNVDLIPK.F R.FQLLEGPPESMGR.G + Oxidation (M) K.SPYLYPLYGLGELPQGFAR.L R.NPYYGGESSSITPLEELYKR.F	22 64 41 26 45
MK01_RAT/P63086	Mitogen-activated protein kinase 1	41648/ 6.50	4(0)/ 8%	79	R.GQVFDVGPR.Y K.ELIFEETAR.F K.LKELIFEETAR.F K.ISPFEHQTYCQR.T	25 21 23 10
STXB1_RAT/P61765	Syntaxin-binding protein 1	67925/ 6.49	2(1)/ 3%	41	R.SQLLILDR.G R.ISEQTYQLSR.W	13 30
ANXA5_RAT/P14668	Annexin A5	35779/ 4.93	3(1)/ 11%	80	K.VLTEIIASR.T R.GTVTDFSGFDGR.A K.GLGTDEDSILNLLTAR.S	22 37 21
TBB2A_RAT/P85108	Tubulin beta-2A chain	50274/ 4.78	4(3)/ 9%	137	R.LHFFMPGFAPLTSR.G R.LHFFMPGFAPLTSR.G + oxidation (M) K.NSSYFVEWIPNNVK.T R.INVYYNEATGGKYVPR.A	46 (11) 57 34
TBB2C_RAT/Q6P9T8	Tubulin beta-2C chain	50225/ 4.79	5(4)/ 11%	188	R.YLTVAAVFR.G R.LHFFMPGFAPLTSR.G R.LHFFMPGFAPLTSR.G + oxidation (M) K.NSSYFVEWIPNNVK.T R.INVYYNEATGGKYVPR.A	55 46 (11) 57 30
TBB3_RAT/Q4QRB4	Tubulin beta-3 chain	50842/ 4.82	2(1)/ 6%	72	R.LHFFMPGFAPLTAR.G + oxidation (M) K.NSSYFVEWIPNNVK.V	18 57
TBA1A_RAT/P68370	Tubulin alpha-1A chain	50788/ 4.94	4(4)/ 13%	246	R.QLFHPEQLITGK.E R.AVFVDLEPTVIDEVR.T R.IHFPLATYAPVISAEK.A R.AVCMLSNTTAIAEAWAR.L	32 100 67 47
TBA1C_RAT/Q6AYZ1	Tubulin alpha-1C chain	50590/ 4.96	4(4)/ 13%	246	R.QLFHPEQLITGK.E R.AVFVDLEPTVIDEVR.T R.IHFPLATYAPVISAEK.A R.AVCMLSNTTAIAEAWAR.L	32 100 67 47
TBA1B_RAT/Q6P9V9	Tubulin alpha-1B chain	50804/ 4.94	4(4)/ 13%	246	R.QLFHPEQLITGK.E R.AVFVDLEPTVIDEVR.T R.IHFPLATYAPVISAEK.A R.AVCMLSNTTAIAEAWAR.L	32 100 67 47
TBA4A_RAT/Q5XIF6	Tubulin alpha-4A chain	50634/ 4.95	4(4)/ 13%	185	R.QLFHPEQLITGK.E R.AVFVDLEPTVIDEIR.N R.IHFPLATYAPVISAEK.A R.AVCMLSNTTAIAEAWAR.L	32 41 67 47
ACTB_RAT/P60711	Actin, cytoplasmic 1	42052/ 5.29	5(4)/ 20%	235	R.AVFPSIVGRPR.H K.IWHHTFYNELR.V K.SYELPDGQVITIGNER.F R.VAPEEHPVLLTEAPLNPK.A K.DLYANTVLSGGTTMYPGIADR.M + Oxidation (M)	35 62 78 49 13
MDHM_RAT/P04636	Malate dehydrogenase, mitochondrial	36117/ 8.93	1(1)/ 4%	33	K.AGAGSATLSMAYAGAR.F + Oxidation (M)	33
ROA2_RAT/A7VJC2	Heterogeneous nuclear ribonucleoproteins A2/B1	37512/ 8.97	3(2)/ 10%	86	R.GGNFGFGDSR.G R.GGGGNFGPGPGSNFR.G K.YHTINGHNAEVR.K	30 32 24
COF1_RAT/P45592	Cofilin-1	18749/ 8.22	2(1)/ 16%	107	R.YALYDATYETK.E K.LTGIKHELQANCYEEVK.D	90 17
DYN1_RAT/P21575	Dynamin-1	97576/ 6.44	2(1)/ 2%	51	K.FTDFEEVR.L K.VLNQQLTNHIR.D	26 25
NFL_RAT/P19527	Neurofilament light polypeptide	61355/ 4.63	5(4)/ 13%	195	R.ALYEQEIR.D R.YLKEYQDLLNVK.M R.SAYSGLQSSSYLMSAR.A + Oxidation (M) R.SAYSSYSAPVSSSLSVR.R R.LSFTSVGSITSGYSQSSQVFGR.S	40 64 44 23 27
NFM_RAT/P12839	Neurofilament medium polypeptide	95848/ 4.77	2(2)/ 3%	83	K.VQSLQDEVAFLR.S R.FSTFSGSITGPLYTHR.Q	55 27
PRDX1_RAT/Q63716	Peroxiredoxin-1	22323/ 8.27	4(2)/ 20%	133	K.IGHPAPSFK.A R.TIAQDYGVLK.A R.LVQAFQFTDK.H R.QITINDLPVGR.S	50 20 39 23
PRDX2_RAT/P35704	Peroxiredoxin-2	21941/ 5.34	1(1)/ 9%	25	K.SLSQNYGVLKNDEGIAYR.G	25
SODM_RAT/P07895	Superoxide dismutase [Mn], mitochondrial	24887/ 8.96	2(1)/ 13%	87	K.AIWNVINWENVSQR.Y K.HHATYVNNLNVTEEK.Y	3 84
GSTP1_RAT/P04906	Glutathione S-transferase P	23652/ 6.89	2(1)/ 13%	47	M.PPYTIVYFPVR.G K.YGTLIYTNYENGKDDYVK.A	47 0
GRP78_RAT/P06761	78 kDa glucose-regulated protein	72473/ 5.07	1(1)/ 2%	29	K.VTHAVVTVPAYFNDAQR.Q	29
HS90A_RAT/ P82995	Heat shock protein HSP 90-alpha	85161/ 4.93	5(1)/ 7%	79	R.RAPFDLFENR.K R.GVVDSEDLPLNISR.E K.HLEINPDHSIIETLR.Q R.NPDDITNEEYGEFYK.S K.KHLEINPDHSIIETLR.Q	15 15 9 10 29
HS90B_RAT/ P34058	Heat shock protein HSP 90-beta	83571/ 4,97	2(0)/ 4%	26	R.GVVDSEDLPLNISR.E R.NPDDITQEEYGEFYK.S	15 11
HSP7C_RAT/P63018	Heat shock cognate 71 kDa protein	71055/ 5.37	4(2)/ 9%	114	K.DAGTIAGLNVLR.I R.TTPSYVAFTDTER.L K.NQVAMNPTNTVFDAKR.L + Oxidation (M) K.TVTNAVVTVPAYFNDSQR.Q	9 66 10 28
ENPL_RAT/Q66HD0	Endoplasmin	92998/ 4.72	4(2)/ 5%	103	R.GLFDEYGSK.K K.FAFQAEVNR.M K.SILFVPTSAPR.G R.FQSSHHSTDITSLDQYVER.M	45 23 30 7
PPIA_RAT/P10111	Peptidyl-prolyl cis-trans isomerase A	18091/ 8.34	4(0)/ 27%	81	K.FEDENFILK.H K.GFGYKGSSFHR.I R.VCFELFADKVPK.T K.TEWLDGKHVVFGK.V	23 23 15 20
			5(5)/ 38%	224	K.FEDENFILK.H K.GFGYKGSSFHR.I R.VCFELFADKVPK.T K.TEWLDGKHVVFGK.V M.VNPTVFFDITADGEPLGR.V	45 54 44 38 48
LDHA_RAT/P04642	L-lactate dehydrogenase A chain	18749/ 8.22	4(2)/ 11%	105	K.LVIITAGAR.Q R.FRYLMGER.L + Oxidation (M)e K.SADTLWGIQK.E K.VTLTPDEEAR.L	30 23 35 17
LDHB_RAT/P42123	L-lactate dehydrogenase B chain	36874/ 5.70	3(2)/ 8%	121	K.IVVVTAGVR.Q R.GLTSVINQK.L K.SADTLWDIQK.D	23 31 67
MDHC_RAT/O88989	Malate dehydrogenase, cytoplasmic	36631/ 6.16	4(2)/ 15%	105	K.GEFITTVQQR.G K.FVEGLPINDFSR.E K.SAPSIPKENFSCLTR.L K.EVGVYEALKDDSWLK.G	37 36 14 18
NDUS2_RAT/Q641Y2	NADH dehydrogenase [ubiquinone] iron-sulfur protein 2, mitochondrial	52927/ 6.52	2(1)/ 7%	56	K.GEFGVYLVSDGSSRPYR.C K.TQPYDVYDQVEFDVPIGSR.G	33 23
DLDH_RAT/Q6P6R2	Dihydrolipoyl dehydrogenase, mitochondrial	54574/ 7.96	3(2)/ 6%	73	R.GIEIPEVR.L K.VGKFPFAANSR.A R.VCHAHPTLSEAFR.E	12 37 27
DHE3_RAT/P10860	Glutamate dehydrogenase 1, mitochondrial	61719/ 8.05	1(1)/ 2%	32	R.DDGSWEVIEGYR.A	32
ATPA_RAT/P15999	ATP synthase subunit alpha, mitochondrial	59831/ 9.22	4(4)/ 9%	226	R.VGLKAPGIIPR.I K.LYCIYVAIGQKR.S R.EAYPGDVFYLRSR.L R.TGAIVDVPVGDELLGR.V	41 33 80 71
			4(2)/ 8%	100	R.VLSIGDGIAR.V K.AVDSLVPIGR.G R.EAYPGDVFYLHSR.L R.ILGADTSVDLEETGR.V	16 33 32 19
QCR1_RAT/Q68FY0	Cytochrome b-c1 complex subunit 1	53500/ 5.57	2(2)/ 5%	118	R.RIPLAEWESR.I K.EVESIGAHLNAYSTR.E	39 81
QCR2_RAT/P32551	Cytochrome b-c1 complex subunit 2, mitochondrial	48423/ 9.16	3(1)/ 7%	72	K.AVAFQNPQTR.I K.EVAEQFLNIR.G K.NALANPLYCPDYR.M	21 21 30
ACON_RAT/Q9ER34	Aconitate hydratase mitochondrial	86121/ 7.87	6(6)/ 9%	300	R.DGYAQILR.D K.EGWFLDIR.V K.SQFTITPGSEQIR.A K.VAMSHFEPSEYIR.Y + oxidation (M) R.NAVTQEFGPVPDTAR.Y R.WVVIGDENYGEGSSR.E	49 48 37 42 51 75
			6(4)/ 9%	175	K.EGWPLDIR.V R.HLGGRAIITK.S K.VAMSHFEPSEYIR.Y + Oxidation (M) R.NAVTQEFGPVPDTAR.Y R.WVVIGDENYGEGSSR.E K.IVYGHLDDPANQEIER.G	33 9 43 44 43 5
EFTU_RAT/P85834	Elongation factor Tu, mitochondrial	49890/ 7.23	1(1)/ 3%	54	R.GITINAAHVEYSTAAR.H	54
SPTA2_RAT/P16086	Spectrin alpha chain	285261/ 5.20	3(0)/ 1%	44	R.EELITNWEQIR.T R.ELPTAFDYVEFTR.S K.HQAFEAELHANADR.I	25 21 6
ACTZ_RAT/P85515	Alpha-centractin	42701/ 6.19	1(1)/ 2%	43	K.YCFPNYVGRPK.H	43
SNAB_RAT/P85969	Beta-soluble NSF attachment protein	33791/ 5.32	2(2)/ 8%	118	K.VAAYAAQLEQYQK.A K.YEEMFPAFTDSR.E + Oxidation (M)	79 39
TKT_RAT/P50137	Transketolase	68342/ 7.23	3(1)/ 8%	67	R.TVPFCSTFAAFFTR.A K.ILATPPQEDAPSVDIANIR.M R.TSRPENAIIYSNNEDFQVGQAK.V	33 24 10

^a^Accession number of Swiss-Prot database.

^b^Sequence coverage.

^c^Protein score: protein overall scores higher than 25 are significant (P < 0.05).

^d^Ion score.

Importantly, all protein assignments listed in this study had a false discovery rate (FDR) of 0%, as estimated by decoy database analysis provided by Mascot. Because FDR is a source of error in protein identification, its estimation is important to validate data. Searches against a composite target-decoy database have been shown to provide an effective way of FDR estimation [24].

The analysis led to the identification of 86 different proteins contained in 97 spots. Figure 1 shows that there were 57 spots in which only one protein was identified. In six spots, 2, 3, or 4 proteins were identified. In four cases, proteins were highly homologous (subtypes of tubulin alpha and beta chains; 14-3-3 protein zeta/delta and theta; heat shock protein HSP 90 alpha and beta) while in two cases, 2 proteins constituting the same spot were not related (heterogeneous nuclear ribonucleoproteins A2/B1 and malate dehydrogenase mitochondrial; glutamate dehydrogenase 1 and dihydrolipoyl dehydrogenase). The fact that their molecular masses and isoelectric points are very similar suggests the occurrence of protein overlapping. It is important to point out that we found 60% of the spots to comprise a single polypeptide chain, agreeing with the analysis of overlapping as a function of protein load [25].

Additional experiments were performed using isoelectric focusing with pH 4–7 strips with the objective of better resolving spots presenting multiple proteins. In the 6 gels performed, 141 ± 1 spots were resolved, of which 50 were cut and digested. The analysis led to the identification of 38 proteins, whose identity confirmed the results obtained with the broad-range strip (data not shown). These findings demonstrate that the use of pH 3–10 IEF strips is appropriate for proteomic studies of rat hypothalamus.

Finally, 17 proteins were identified in more than one spot, for example, the enzymes fructose-bisphosphate aldolase C (P09117, 2 spots), glyceraldehyde-3-phosphate dehydrogenase (P04797, 3 spots), and dihydropyrimidinase-related protein 2 (P47942, 3 spots). The presence of one protein in multiple spots has been suggested to reflect isoforms or post-translational modifications. Creatine kinase B-type (P07335), an enzyme that plays a role in cell energy metabolism, is represented in two spots differing in isoelectric points. This enzyme is also present in different spots of 2-D gels of crude microtubule preparations from rat brains, the isoelectric variants corresponding to a phosphorylated and a non-phosphorylated form [26].

**Figure 2:**
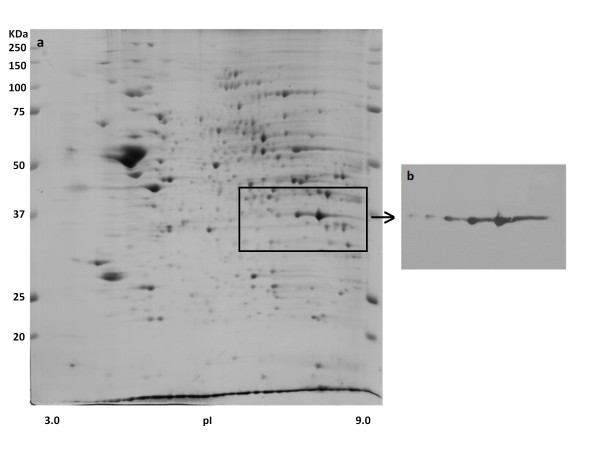
**Western blot confirmation of protein identity.** 2-D gel of rat hypothalamus stained with Coomassie Blue G-250 **(a)**. Immunobloting of glyceraldehyde-3-phosphate dehydrogenase **(b)**.

In order to confirm protein identification, we performed one additional experiment using Western blot for the protein glyceraldehyde-3-phosphate dehydrogenase. Figure 2, showing high binding of the specific antibody to the three analysed spots indicate the accuracy of the protein identification in the present study.

Approximately 75% of the identified proteins had molecular masses in the 30–100 KDa range and 79% had pI values between 4 and 8. Thus, we were able to identify only four low molecular weight proteins (under 20 kDa) (l-lactate dehydrogenase A chain, P04642; nucleoside diphosphate kinase B, P19804; cofilin-1, P45592; peptidyl-prolyl cis-trans isomerase A, P10111) and only one protein with a molecular weight above 150 kDa (spectrin alpha-chain, P16086). This agrees with the low ability of 2-DE protein separation to resolve proteins in upper and lower limits of molecular mass range [27].

**Figure 3:**
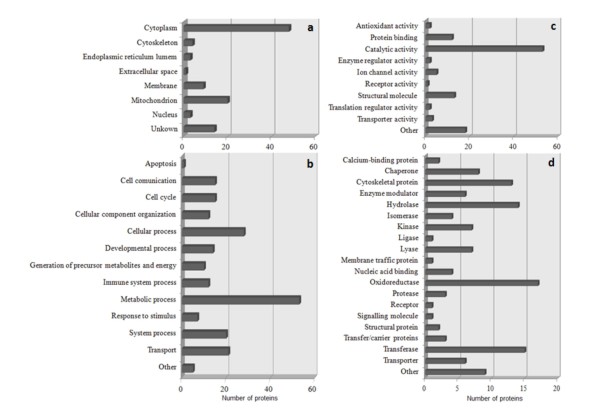
**Protein Ontology.** Identified proteins were classified according to their subcellular location **(a)**, biological processes **(b)**, molecular function **(c)**, and protein class **(d)**.

The Swiss-Prot database was used to classify proteins according to their subcellular location and, as expected, most of the identified proteins are cytoplasmatic, as shown in Figure 3a. Although both the chaotropic agent thiourea and the zwitterionic detergent CHAPS were used in the extraction buffer to maximize membrane protein solubility [20], only 10% of the identified proteins were membrane proteins. This low recovery has been associated with the poor solubilization of hydrophobic molecules in 2-DE technique [28,29].

Gene Ontology analysis of identified proteins was performed using the bioinformatics tool PANTHER [30] (http://www.pantherdb.org/). In this way, proteins were categorized according to biological processes (Figure 3b), molecular function (Figure 3c) and protein class (Figure 3d). An additional file shows the classification of each protein in more detail (see Additional file 1).

The majority of the identified proteins were classified as participating in metabolic processes (Figure 3b), consistent with the finding of a large number of proteins with catalytic activity (Figure 3c). Oxidoreductases, transferases, and hydrolases were the main protein classes identified in this study (Figure 3d).

Genes encoding some proteins identified in this study have been related to obesity development. When gene expression profile was performed in obese subjects, genes involved in NADH dehydrogenase (ubiquinone) activity, glutamate dehydrogenase activity and glutamate decarboxylase activity, among others, were up-regulated in comparison to normal lean individuals [31].

GAD2 polymorphisms are believed to be associated with disrupted eating behavior and obesity risk in women [32]. The GAD2 gene encodes the enzyme glutamate decarboxylase 2 (Q05683), which catalyzes glutamate decarboxylation to γ-aminobutyric acid (GABA). According to the authors, GAD2 polymorphisms could modulate increases in hypothalamic GABA concentration, enhancing the orexigenic effect of neuropeptide Y (NPY). Moreover, GAD2 has been suggested to participate in complex polygenic mechanisms linking birth weight to further risk for metabolic diseases [33].

Excessive superoxide production has been related to obesity and several other chronic diseases. An association between obesity and manganese-dependent superoxide dismutase gene polymorphism has been reported in elderly subjects. A possible biological explanation of this association could be a chronic state of superoxide enzyme (P07895) imbalance present in subject carriers of gene polymorphism, which could affect differential metabolic pathways contributing to obesity development [34].

With the use of proteomic approach in rat hypothalamic tissue, it has recently been reported that perinatal undernutrition modulated numerous metabolic pathways, resulting in alterations of hypothalamic energy supply. In the 12–16 day-old male offspring, proteins related to energy-sensing, redox status, amino acid pathways and neurodevelopment, including fructose bisphosphate aldolase C (P09117), malate dehydrogenase cytoplasmic (O88989), and dihydropyrimidinase related protein 2 (P47942) were affected [35]. We have previously shown that female adult rats exposed to intrauterine food restriction had obesity and defects in hypothalamic systems involved in feeding regulation [36,37]. Importantly, we are currently applying the present protocol of hypothalamic proteomic analysis to further characterize the late consequences of intrauterine food restriction.

In another study in the nutrition field, cows subjected to energy restriction for 60 hours showed altered expression of nine hypothalamic proteins that are potential candidate molecules involved in maintaining energy homeostasis, such as ubiquitin carboxyl-terminal hydrolase L1 (Q00981) [38]. Furthermore, chronic circadian desynchronization has led to increased food intake, weight gain and fat mass in rats, and affected the expression of five hypothalamic proteins: fructose-bisphosphate aldolase A (P05065), aconitase 2, GABA aminotransferase, aldehyde dehydrogenase family 1 member B1 and VDAC1 (Q9Z2L0) [8].

## Conclusion

In summary, the methods described herein allowed identification of 86 hypothalamic proteins, some of which have already been found to be altered in obesity. There have been relatively few analyses of hypothalamic proteomes of male *Wistar* rats and the current identification contributes to the building of a database for this important neuroendocrine structure. These results will serve as a reference database for studies testing the effects of dietary manipulations on hypothalamic proteome. We trust that these experiments will lead to important knowledge on protein targets of nutritional variables potentially able to affect the complex central nervous system control of energy homeostasis.

## Methods

### Animals and sample preparation

All procedures were in compliance with guidelines of Committee on Research Ethics of Federal University of São Paulo. Male *Wistar* were kept under controlled conditions of temperature and lighting (lights on from 6:00 to 18:00 h). Four- month-old rats were killed by decapitation, the skull was rapidly opened and the hypothalami removed and immediately homogenized in 1 mL of extraction buffer. The hypothalamus was dissected having the thalamus as the dorsal limit, with the rostral and caudal limits being the optic chiasm and the mammillary bodies, respectively [39].

In order to determine the optimal conditions for proteome analysis of rat hypothalamus, a series of tests were performed.2-D gels of samples prepared with five different extraction and rehydration buffers were analysed for spot counting and resolution. All buffers contained complete Mini Protease Inhibitor Cocktail Tablets (Roche Diagnostics, Germany), added immediately before use. Each rat sample was analyzed individually, with no tissue pooling. After sample homogenization, lysates were centrifuged at 14.000 rpm for 30 minutes; supernatants were collected and stored at - 80°C until use. Table 1 summarizes buffers composition and the procedures used for sample preparation.

### Protein assay

Protein concentration of supernatants was determined using 2-D Quant Kit (GE Healthcare, USA) and bovine albumin as standard, according to manufacturer's recommendations. Protein concentrations were typically between 3 and 4.5 mg/mL.

### Protein precipitation

Aliquots of 750 μg of protein were precipitated with a solution of 35% KCl, 44% chloroform, and 21% methanol (v/v). The mixture was homogenized by vortex mixing and centrifuged at 14.000 rpm and 4°C for 15 min. The pellet was recovered and air-dried.

### Two-dimensional gel electrophoresis and image analysis

For isoelectric focusing (IEF), the pellet was dissolved in 500 μL of rehydration buffer. When protein precipitation was not performed (buffers 1 and 2, please see Table 1), the rehydration solution was added to the sample, to a final volume of 500μL. IEF was carried out on a Protean IEF cell (Bio-Rad, USA) using immobiline dry strips (18 cm linear gradient, pH 3–10 and pH 4–7) previously rehydrated for 12-14 h. IEF was performed with the current limit set at 50 mA per IPG strip with the following conditions at 18°C: 1000 V for 30 minutes, 2000 V for 1 hour, 4000 V for 1 hour, 8000 V for one hour, 8000 V until 40000 Vh.

After focusing, strips were equilibrated for 15 min in buffer containing 6 M urea, 50 mM Tris base pH 8.8, 34% (v/v) glycerol, 2% (w/v) SDS (Sodium Dodecyl Sulfate), and 1% (w/v) DDT, followed by an additional 15 min in the same buffer containing 2.5% (w/v) iodocetamide instead of DTT. Strips were then loaded onto 12% SDS-polyacrylamide gels. After running in Protean II Multi-Cell (Bio-Rad, USA), at 50 mA per gel for 5 hours, the gels were stained for 48 h with Coomassie Blue G-250 (Bio-Rad, USA). Stained gels were scanned (GS-710 Calibrated Imaging Densitometer) and analyzed using PDQuest Image Analysis Software version 7.2 (Bio-Rad, USA).

To construct a two-dimensional gel electrophoresis profile of rat hypothalamus proteins, six different rats were used and each hypothalamus was analysed separately, in two independent experiments. Each analyzed spot was present in all six gels.

### Matrix-assisted laser desorption ionization time-of-flight mass spectrometry

The selected spots were automatically excised, destained and digested, using Xcise^TM^ spot picker (Shimadzu Biotech, Japan), as reported elsewhere [40]. Briefly, excised spots were destained in 100 mM ammonium bicarbonate and 50% acetonitrile (1:1) and digestion was performed in 30 μL of 10 ng/μL trypsin (Sigma Aldrich, Germany) in 25 mM ammonium bicarbonate, overnight at 34°C. Digested samples were desalted using μC_18_ Zip Tips (Millipore, Ireland). One microliter of sample was mixed with matrix solution (10 mg/mL α-cyano-4 hydroxycinnamic acid in 70% acetonitrile/0.1% trifluoroacetic acid), applied on the spectrometer plate and air dried at room temperature.

MALDI-TOF/TOF MS was performed using an Axima Performance ToF-ToF, (Kratos-Shimadzu Biotech, UK) mass spectrometer. The instrument was externally calibrated with [M + H]^+^ ions of bradykinin 1–7 fragment (757.4 Da), human angiotensin II (1046.54 Da), P_14_R synthetic peptide (1533.86 Da), and human ACTH 18–39 fragment (2465.20 Da). Following MALDI MS analysis, MALDI MS/MS was performed on 7 most abundant ions from each spot.

MASCOT (Matrix Science, UK) server was used to search Swiss-Prot protein database (http://www.expasy.ch/sprot/). The following parameters were used in this search: no restrictions on protein molecular weight, trypsin digest with one missing cleavage, monoisotopic mass, taxonomy limited to *Rattus*, carbamidomethylation of cysteine as fixed modification, possible oxidation of methionine and tryptophan, peptide mass tolerance of 0.5 Da, fragment mass tolerance of 0.8 Da, and peptide charge +1. False discovery rate (FDR) assessment was estimated using Mascot decoy database approach and only proteins identified with 0% FDR were included in the results.

Protein matching probabilities were determined using MASCOT protein scores, with identification confidence indicated by the number of matching and the coverage of protein sequence by the matching peptides. The presence of at least one peptide with significant ion score was required for positive protein identification. Only statistically significant MASCOT score results (p < 0.05) were included in the analysis.

### Western blot procedures

For confirmation of protein identity, two 2-D gels were performed in identical conditions as those described above, with protein extracted from one rat hypothalamus. One of the gels was stained with Coomassie Blue G-250 while the other was cut and had its proteins transferred to a nitrocellulose membrane, as previously described [41]. After blockage, the membrane was incubated with glyceraldehyde-3-phosphate dehydrogenase antibody (GAPDH, sc-25778, Santa Cruz Biotechnology, USA) followed by secondary anti-rabbit antibody and revealed by chemiluminescense.

## Competing interests

The authors declare that they have no competing interests.

## Authors' contributions

APP contributed to the overall conception and design of the project and carried out the experiments, analysis and interpretation of the data, and preparation of the manuscript. RLHW contributed to the overall conception and design of the project and carried out the experiments, analysis and interpretation of the data. KTA contributed to the overall conception and design of the project and carried out the experiments. MMT, MCCA, and DE carried out experiments. JDP, MMS and MLL contributed with technical support and to the analysis of the data. CMON and LMO contributed with critical discussions. JCR performed all mass spectrometry analysis. DEC contributed to the overall conception and design of the project and to data analysis and interpretation. EBR conceived the study, and participated in its design and coordination, data analysis and interpretation, and preparation of the manuscript. All authors have read and approved the final manuscript.

## Supplementary Material

Additional file 1 Classification of the identified proteins of the rat hypothalamus. The data lists the classification of the hypothalamic proteins identified here using Panther Classification System (http://www.pantherdb.org/). Click here for file
