# Supplementary material for: Proteomic profiling of the rat hypothalamus
Source: Proteome Sci. 2012 Apr 20;10:26. doi: 10.1186/1477-5956-10-26 (PMC3441799; doi:10.1186/1477-5956-10-26)
Supplement: Additional file 1 — Classification of the identified proteins of the rat hypothalamus. The data lists the classification of the hypothalamic proteins identified here using Panther Classification System (http://www.pantherdb.org/). [file 1477-5956-10-26-S1.pdf]

Full name	Cellular location	Biological processes	Molecular function	Protein class
Glyceraldehyde-3-phosphate dehydrogenase	Cytoplasm	Metabolic process	Catalytic activity	Oxidoreductase
Succinate-semialdehyde dehydrogenase, mitochondrial	Mitochondrion	Metabolic process	Catalytic activity	Oxidoreductase
Glutamate dehydrogenase 1, mitochondrial	Mitochondrion	Metabolic process	Catalytic activity	Oxidoreductase
Alcohol dehydrogenase [NADP+]	-	Metabolic process	Catalytic activity<br>Ion channel activity	Oxidoreductase
2',3'-cyclic-nucleotide 3'-phosphodiesterase	Membrane	Metabolic process	Catalytic activity	Hydrolase
Dihydropyrimidina-se-related protein 2	Cytoplasm. Tightly but noncovalently associate with membranes	Metabolic process	Catalytic activity	Hydrolase
Dihydropyrimidina-se-related protein 5	Cytoplasm	Metabolic process	Catalytic activity	Hydrolase
Ubiquitin carboxyl-terminal hydrolase isozyme L1	Cytoplasm	Metabolic process	Catalytic activity	Hydrolase<br>Protease
ATP synthase subunit gamma, mitochondrial	Mitochondrion	Metabolic process<br>Transport	Catalytic activity<br>Transporter activity	Hydrolase<br>Transporter
V-type proton ATPase subunit E 1	-	Metabolic process<br>Transport	Catalytic activity<br>Transporter activity	Hydrolase<br>Transporter
4-aminobutyrate aminotransfe-rase, mitochondrial	Mitochondrion	Metabolic process<br>System process	Catalytic activity	Transferase
Aspartate aminotransferase, cytoplasmic	Cytoplasm	Metabolic process	Catalytic activity	Transferase
Nucleoside diphosphate kinase B	Cytoplasm. Cell membrane	Metabolic process	Catalytic activity	Kinase<br>Transferase
Pyruvate kinase isozymes M1/M2	Cytoplasm. Nucleus	Metabolic process	Catalytic activity	Kinase<br>Transferase
Hexokinase-1	Mitochondrion	Metabolic process	Catalytic activity	Kinase<br>Transferase
Phosphoglycerate kinase 1	Cytoplasm	Metabolic process	Catalytic activity	Kinase<br>Transferase
Creatine kinase B-type	Cytoplasm	Metabolic process<br>System process	Catalytic activity	Kinase<br>Transferase
Creatine kinase U-type	Mitochondrion	Metabolic process<br>System process	Catalytic activity	Kinase<br>Transferase
Gamma-enolase	Cytoplasm. Cell membrane	Metabolic process	Catalytic activity	Lyase
Fructose-bisphosphate aldolase C	Cytoplasm	Metabolic process	Catalytic activity	Lyase

Alpha-enolase	Cytoplasm. Cell membrane	Metabolic process	Catalytic activity	Lyase
Fructose-bisphosphate aldolase A	-	Metabolic process	Catalytic activity	Lyase
Glutamate decarboxylase 2	Cytoplasm	Metabolic process	Catalytic activity	Lyase
Glycogen phosphorylase, brain form (fragment)	-	Metabolic process	Catalytic activity	Isomerase<br>Transferase
Triosephosphate isomerase	-	Metabolic process	Catalytic activity	Isomerase
Glutamine synthetase	Cytoplasm. Mitochondrion	Metabolic process	Catalytic activity	Ligase
Calreticulin	Endoplasmic reticulum lumen	Metabolic process	Protein binding	Calcium-binding protein
Secernin-1	Cytoplasm	Metabolic process	-	-
Protein IMPACT	-	Metabolic processes	-	-
Phosphoglycerate mutase 1	-	Metabolic process	Catalytic activity	-
Voltage-dependent anion-selective channel protein 1	Mitochondrion. Cell membrane	Transport	Ion channel activity	Transporter
Voltage-dependent anion-selective channel protein 2	Mitochondrion	Transport	Ion channel activity	Transporter
Serum albumin	Extracellular space	Transport	-	Transfer/carrier protein
Vesicle-fusing ATPase	Cytoplasm	Cell comunication<br>Cellular process<br>System process<br>Transport	Catalytic activity	Hydrolase
Elongation factor 1-gamma	-	Cell communication<br>Cellular process<br>Immune system process<br>Metabolic process<br>Response to stimulus<br>Transport	Protein binding<br>Catalytic activity<br>Ion channel activity<br>Translation regulator	Cytoskeletal protein<br>Isomerase<br>Nucleic acid binding<br>Oxidoreductase<br>Signalling molecule<br>Transferase<br>Transporter
14-3-3 protein gamma	Cytoplasm	Cell communication<br>Cell cycle<br>Cellular process	-	Chaperone
14-3-3 protein épsilon	Cytoplasm	Cell communication<br>Cell cycle<br>Cellular process	-	-
14-3-3 protein beta/alpha	Cytoplasm	Cell communication<br>Cell cycle<br>Cellular process	-	Chaperone
14-3-3 protein zeta/delta	Cytoplasm	Cell communication<br>Cell cycle<br>Cellular process	-	Chaperone
14-3-3 protein theta	Cytoplasm	Cell communication<br>Cell cycle<br>Cellular process	-	Chaperone

Phosphatidylethanolamine-binding protein 1	Cytoplasm. Membrane	Cell comunication<br>Cellular process	Protein binding<br>Enzyme regulator activity	Enzyme modulator<br>Transfer/carrier protein
Elongation factor 2	Cytoplasm	Cell comunication<br>Cellular process<br>Metabolic process	Protein binding<br>Catalytic activity	Enzyme modulator<br>Hydrolase<br>Nucleic acid binding<br>Transferase
Guanine nucleotide-binding protein G(o) subunit alpha	-	Cell comunication<br>Cellular process	Protein binding<br>Catalytic activity	Enzyme modulator
Guanine nucleotide-binding protein G(I)/G(S)/G(T) subunit beta-1	Plasma membrane	Cell comunication<br>Cellular process<br>System process	Protein binding<br>Catalytic activity	Enzyme modulator<br>Hydrolase
Rab GDP dissociation inhibitor alpha	Cytoplasm	Cell comunication<br>Cellular process<br>System process<br>Transport	Protein binding<br>Catalytic activity<br>Enzyme regulator activity	Enzyme modulator<br>Transferase
Mitogen-activated protein kinase 1	-	Cell communication<br>Cell cycle<br>Cellular process<br>Developmental process<br>Immune system process<br>Metabolic process<br>Response to stimulus	Catalytic activity	Kinase<br>Transferase
Syntaxin-binding protein 1	Cytoplasm. Membrane	Cell communication<br>Cellular process<br>System process<br>Transport	-	Membrane traffic protein
Annexin A5	Plasma membrane	Cell comunication<br>Cellular process<br>Metabolic process<br>Transport	Protein binding	Calcium-binding protein<br>Transfer/carrier protein
Tubulin beta-2A chain	Cytoplasm	Cell cycle<br>Cellular component organization<br>Cellular process<br>Developmental process<br>Transport	Structural molecule	Cytoskeletal protein
Tubulin beta-2C chain	Cytoplasm	Cell cycle<br>Cellular component organization<br>Cellular process<br>Developmental process<br>Transport	Structural molecule	Cytoskeletal protein
Tubulin beta-3 chain	Cytoplasm	Cell cycle<br>Cellular component organization<br>Cellular process<br>Developmental process<br>Transport	Structural molecule	Cytoskeletal protein

Tubulin alpha-1A chain	Cytoplasm	Cell cycle<br>Cellular component organization<br>Cellular process<br>Developmental process<br>Transport	Structural molecule	Cytoskeletal protein
Tubulin alpha-1C chain	Cytoplasm	Cell cycle<br>Cellular component organization<br>Cellular process<br>Developmental process<br>Transport	Structural molecule	Cytoskeletal protein
Tubulin alpha-1B chain	Cytoplasm	Cell cycle<br>Cellular component organization<br>Cellular process<br>Developmental process<br>Transport	Structural molecule	Cytoskeletal protein
Tubulin alpha-4A chain	Cytoplasm	Cell cycle<br>Cellular component organization<br>Cellular process<br>Developmental process<br>Transport	Structural molecule	Cytoskeletal protein
Actin, cytoplasmic 1	Cytoplasm	Cell cycle<br>Cellular component organization<br>Cellular process<br>Developmental process<br>Transport	Structural molecule	Cytoskeletal protein
Heterogeneous nuclear ribonucleoproteins A2/B1	Nucleus	Cell cycle<br>Cellular process<br>Developmental process<br>Metabolic process<br>Reproduction<br>System process	Protein binding<br>Catalytic activity	Nucleic acid binding
Cofilin-1	Nucleus	Cellular component organization<br>Cellular process<br>Developmental process<br>Metabolic process	Protein binding<br>Structural molecule	Cytoskeletal protein
Dynamin-1	Cytoplasm	Cellular component organization<br>Cellular process<br>Developmental process<br>Transport	Protein binding<br>Catalytic activity<br>Structural molecule	Cytoskeletal protein<br>Enzyme modulator<br>Hydrolase
Neurofilament light polypeptide	Cytoskeleton	Cellular component organization<br>Cellular process<br>Developmental process	Structural molecule	Cytoskeletal protein<br>Structural protein

Neurofilament medium polypeptide	Cytoskeleton	Cellular component organization<br>Cellular process<br>Developmental process	Structural molecule	Cytoskeletal protein<br>Structural protein
Peroxiredoxin-1	Cytoplasm	Immune system process<br>Metabolic process	Antioxidant activity<br>Catalytic activity	Oxidoreductase
Peroxiredoxin-2	Cytoplasm	Immune system process<br>Metabolic process	Antioxidant activity<br>Catalytic activity	Oxidoreductase
Superoxide dismutase [Mn], mitochondrial	Mitochondrion	Immune system process<br>Metabolic process	Catalytic activity	Oxidoreductase
Glutathione S-transferase P	-	Immune system process<br>Response to stimulus	Catalytic activity	Transferase
78 kDa glucose-regulated protein	Endoplasmic reticulum lumen	Immune system process<br>Metabolic process<br>Response to stimulus	-	Chaperone
Heat shock protein HSP 90-alpha	Cytoplasm	Immune system process<br>Metabolic process<br>Response to stimulus	-	Chaperone
Heat shock protein HSP 90-beta	Cytoplasm	Immune system process<br>Metabolic process<br>Response to stimulus	-	Chaperone
Heat shock cognate 71 kDa protein	Cytoplasm	Immune system process<br>Metabolic process<br>Response to stimulus	-	Chaperone
Endoplasmin	Endoplasmic reticulum lumen	Immune system process<br>Response to stimulus	-	Chaperone
Peptidyl-prolyl cis-trans isomerase A	Cytoplasm	Immune system process<br>Metabolic process<br>Transport	Catalytic activity	Isomerase
L-lactate dehydrogenase A chain	Cytoplasm	Generation of precursor metabolites and energy<br>Metabolic process	Catalytic activity	Oxidoreductase
L-lactate dehydrogenase B chain	Cytoplasm	Generation of precursor metabolites and energy<br>Metabolic process	Catalytic activity	Oxidoreductase

Malate dehydrogenase, mitochondrial	Mitochondrion	Generation of precursor metabolites and energy<br>Metabolic process	Catalytic activity	Oxidoreductase
Malate dehydrogenase, cytoplasmic	Cytoplasm	Generation of precursor metabolites and energy<br>Metabolic process	Catalytic activity	Oxidoreductase
NADH dehydrogenase [ubiquinone] iron-sulfur protein 2, mitochondrial	Mitochondrion	Generation of precursor metabolites and energy	Catalytic activity	Oxidoreductase
Dihydrolipoyl dehydrogenase, mitochondrial	Mitochondrion	Apoptosis<br>Generation of precursor metabolites and energy<br>Immune system process<br>Metabolic process	Catalytic activity	Oxidoreductase
ATP synthase subunit alpha, mitochondrial	Mitochondrion	Generation of precursor metabolites and energy<br>Metabolic process<br>Transport	Protein binding<br>Catalytic activity<br>Ion channel activity<br>Receptor activity<br>Transporter activity	Hydrolase<br>Nucleic acid binding Receptor<br>Transporter
Cytochrome b-c1 complex subunit 1	Mitochondrion	Generation of precursor metabolites and energy<br>Metabolic process	Catalytic activity	Hydrolase<br>Oxidoreductase<br>Protease
Cytochrome b-c1 complex subunit 2, mitochondrial	Mitochondrion	Generation of precursor metabolites and energy<br>Metabolic process	Catalytic activity	Hydrolase<br>Oxidoreductase<br>Protease
Aconitate hydratase mitochondrial	Mitochondrion	Generation of precursor metabolites and energy<br>Metabolic process	Catalytic activity	Lyase
Elongation factor Tu, mitochondrial	Mitochondrion	-	Structural molecule<br>Translation regulator	-
Spectrin alpha chain	Cytoplasm. Cytoskeleton	-	-	-
Alpha-centractin	Cytoplasm. Cytoskeleton	-	-	-
Beta-soluble NSF attachment protein	Membrane	-	-	-
Transketolase	-	-	Catalytic activity	Lyase<br>Oxidoreductase<br>Transferase
